# Challenges of Organ Shortage for Transplantation: Solutions and Opportunities

**Published:** 2014-08-01

**Authors:** R. F. Saidi, S. K. Hejazii Kenari

**Affiliations:** Division of Organ Transplantation, Department of Surgery, Alpert Medical School of Brown University, Providence, RI, USA

**Keywords:** Organ transplantation, Tissue donor, Tissue and organ procurement, Awareness, Living donors

## Abstract

Organ shortage is the greatest challenge facing the field of organ transplantation today. A variety of approaches have been implemented to expand the organ donor pool including live donation, a national effort to expand deceased donor donation, split organ donation, paired donor exchange, national sharing models and greater utilization of expanded criteria donors. Increased public awareness, improved efficiency of the donation process, greater expectations for transplantation, expansion of the living donor pool and the development of standardized donor management protocols have led to unprecedented rates of organ procurement and transplantation. Although live donors and donation after brain death account for the majority of organ donors, in the recent years there has been a growing interest in donors who have severe and irreversible brain injuries but do not meet the criteria for brain death. If the physician and family agree that the patient has no chance of recovery to a meaningful life, life support can be discontinued and the patient can be allowed to progress to circulatory arrest and then still donate organs (donation after circulatory death). Increasing utilization of marginal organs has been advocated to address the organ shortage.

## Introduction

The greatest challenge facing the field of organ transplantation today is increasing the number of allografts available for transplant. Organ transplantation has proven to be highly effective in the treatment of various forms of end-stage organ failure. Increased public awareness, improved efficiency of the donation process, greater expectations for transplantation, expansion of the living donor pool and the development of standardized donor management protocols have led to unprecedented rates of organ procurement and transplantation. Herein, we focus on donation after circulatory death (DCD) and expanded criteria donors (ECDs).

**THE PROBLEM OF ORGAN SHORTAGE**

Organ transplantation is unquestionably the preferred therapy for most patients with end-stage organ failure since both survival and quality of life are superior in allograft recipients compared to similar patients who without transplantation [1]. As outcomes of transplantation have improved, the number of transplant candidates listed for deceased donor transplantation has increased dramatically over the years. One of the main strategies to address the discrepancy between supply and demand in organ transplantation is expansion of the deceased donor kidney pool utilizing ECD and DCD donors [1–3]. This has been a major focus of the US Department of Health and Human Services organ donation breakthrough collaborative, which was initiated in 2003, with the objective of increasing access to transplantable organs.

In 2009, 50,463 patients were added to transplantation wait list, while 28,463 patients received organ transplantation and 6683 patients died while waiting for a suitable organ [4]. A variety of approaches have been implemented to expand the organ donor pool including increased live donation, a national effort to expand deceased donor donation, split organ donation, paired donor exchange, national sharing models, and greater utilization of ECDs [5,6]

In 2008, more than 28,000 patients received organ transplants from more than 14,000 deceased and live donors in the USA [4,7–13]. Despite the worthy effort of the Organ Donation and Transplant Collaborative and the marked increase in the number of deceased donors early in the effort, the number of deceased donors rose by a total of only 67 from 2006 to 2007 [5]. A recent study showed that the number of living donors has decreased since 2004 and donation after brain death (DBD) also decreased since 2006. This decline can be attributed to increases in the number and percentage of ECDs and DCDs [14]. The shift in the distribution of recovered kidneys from standard criteria donor (SCD) to ECD and DCD impacts utilization, since DCD and ECD kidneys have higher rates of discard [5]. The observed increase in DCD also explains, in part, the fewer organs per donor that are recovered and transplanted overall [6,15,16]. 

**DONATION AFTER CIRCULATORY DEATH**

Although live donors and DBD account for the majority of organ donors, in the recent years there has been a growing interest in donors who have severe and irreversible brain injuries but do not meet the criteria for brain death. If the physician and family agree that the patient has no chance of recovery to a meaningful life, life support can be discontinued and the patient can be allowed to progress to circulatory arrest and then still donate organs (DCD). In the past 10 years, the number of deceased organ donors nationally has increased modestly, whereas DCD has increased 10-fold with over 900 cases of DCD reported in 2009 [7,17,18]. 

**Figure 1:**
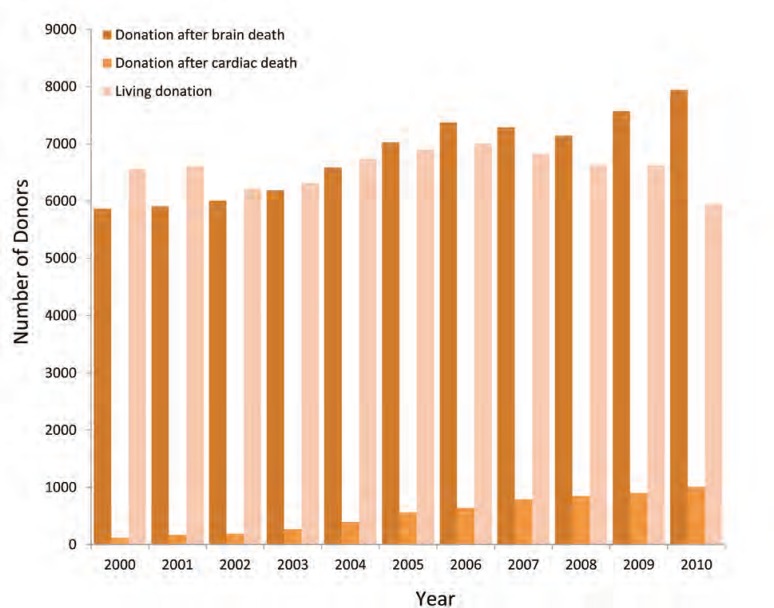
The number of organ donors in the USA (2001–2010).

Consistent with the goals set by Health Resources and Services Administration for DCD development, the percentage of donors from DCD continues to increase. There has been a significant increase in the percentage of donors that are categorized as DCD from 8% in 2006 to 9.8% in 2007, and the number and percentages of DCD liver and kidney transplants continue to increase substantially [6–10,15,17–22]. In a recent study, we examined the pattern of donation and utilization in the USA using Organ Procurement and Transplantation Network/United Network for Organ Sharing (OPTN/UNOS) database of individuals who were consented for and progressed to organ donation between January 2001 and December 2010 (Fig 1). We encountered parallel changes in this study with increasing the number of DCD donors from 3.5% in era-1 (2001–2005) to 9.3% in era-2 (2006–2010) [14]. On the other hand, we noted the decrease in living donation. Although the total number of deceased donors did increase 25% from era-1 to era-2, the number of DBD donors was noted to have peaked in 2006 constantly decreased since. The main reason for the increase in the number of deceased donors was the rapid expansion of the DCD group which rose 230% when comparing era-1 (n=1135) with era-2 (n=3748). At the same time, the number of DBD donors increased by only 17% when comparing era-1 and -2 [14]. Whether this represents addition of donors who would not have ever progressed to brain death or an exchange for DCD in cases that would have previously followed a DBD pathway still remains uncertain. If the latter, this may indicate the occurrence of a change in clinical practice in which withdrawal of support is offered earlier in the patient’s course, before brain death has occurred. Saidi, *et al* [16], identified a significant change in resuscitative practices over time, with a striking rise in new surgical interventions such as craniostomy, craniotomy, cooling, *etc*, that have the potential to intercede in the progression to brain death. These interventions were strongly associated with intent to donate via DCD. The lesser likelihood of making the diagnosis of brain death in these patients provides a plausible explanation for at least part of the stagnant growth of DBD compared with DCD in the national data. 

**Figure 2:**
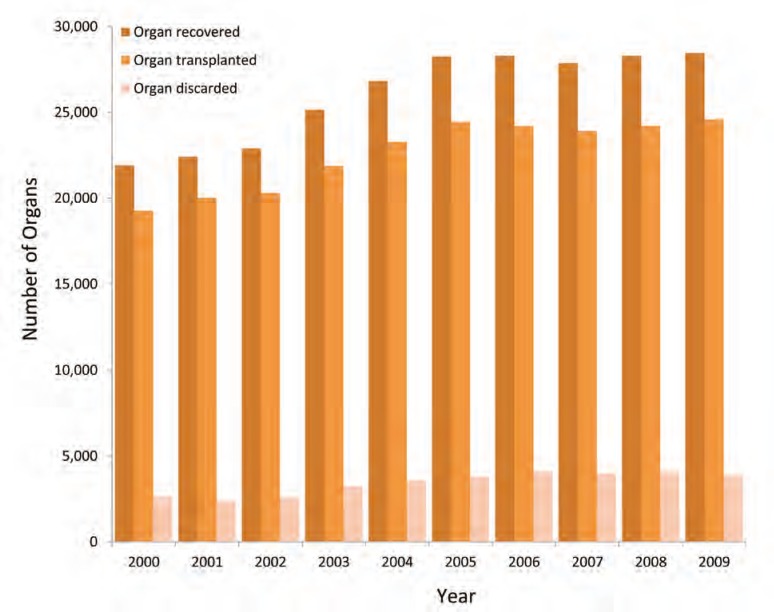
Pattern of deceased organs recovered, transplanted and discarded in the USA (2001–2010).

As a result of increasing utilization of DCD donors, more donors with comorbidities and elderly donors, we also noted a dramatic increase in the discard rates (Fig 2). The overall discard rate increased from 13,411 (11.5%) in era-1 to 19,516 (13.7%) in era-2. This increase in discards was especially prominent in the DCD group which rose from 440 (20.9%) in era-1 to 2,089 (24.9%) in era-2 [14]. The discard rate for DCD livers and kidneys increased. We noted 78% increase in the discard rate of DCD livers and 1210% for discarded DCD kidneys. Although, the discard rates for DBD livers and kidneys remained stable [14].

The new surgical interventions mentioned above, were strongly associated with intent to donate via DCD (*ie*, including potential DCD who did progress to donation in a timely fashion). The lesser likelihood of making the diagnosis of brain death in these patients provides a plausible explanation for at least part of the stagnant growth of DBD compared with DCD [16]. This observation suggests that in addition to implementation of measures intended to be life-saving by preventing herniation, there has been greater adoption of DCD donation in general. Whether this represents addition of donors who would not have ever progressed to brain death or an exchange for DCD in those who would have previously followed a DBD pathway, remains uncertain. If the latter, this may indicate the occurrence of a change in clinical practice in which withdrawal of mechanical, ventilated or organ-perfusion support is offered earlier in the patient’s course, before brain death has occurred. 

Certainly, from the ICU perspective, DCD may be consistent with conscientious and appropriate medical care rather than awaiting a brain death diagnosis in patients with unrecoverable neurological compromise, both saving valuable ICU resources and avoiding unnecessary patient and family suffering. On the other hand, DCD and DBD have markedly different impact in their yield of life saving organs for transplant; this is the consequence of a number of factors. The first is that once consent to donate is established, donation is almost certain for DBD, however, on average 30% of intended DCD do not progress to death in a timely manner after withdrawal of mechanical, ventilated or organ-perfusion support and therefore, result in no organs for transplant. Of even greater magnitude, is the fact that even when donation does occur, on average fewer organs are recovered and transplanted from DCD than DBD. Inspection of UNOS data reveals that nationally in 2009, an average of 3.6 organs were recovered from DBD donors compared to 2.5 organs from DCD; the consented DCD donors that did not progress were not considered. In addition, 3.1 organs were transplanted form DBD donors compared to 1.9 from DCD. On average per 100 donors, DCDs donate 20 less kidney (170 *vs* 190), 40 less liver (40 *vs* 80), and five less pancreas (2 *vs* 7) when compare to DBDs [16].

It should also be noted that our intent is not to challenge the standard of care or ICU management of patients with severe head injury; rather, we have attempted to clarify and recognize the impact these new therapies and practice shifts have on the opportunity for organ recovery from deceased donors. We do recognize however, that there may be specific cases in which there exists a choice to withdraw mechanical, ventilated or organ-perfusion support immediately or to determine if the potential donor will progress to brain death in a timely fashion. In such cases, if the dying patient had expressed a premortem intent to be an organ donor to help others, and if the ability to make the diagnosis of brain death may be imminent, we suggest that it may be appropriate to include in the end-of-life discussion with the next-of-kin the implications of withdrawal of mechanical, ventilated or organ-perfusion support on the nature and magnitude of the gift that was intended by their loved one. We fully understand that organ donation might not be the first or foremost issue on the mid of family or intensive care physicians. However, after the decision to offer withdrawal of mechanical, ventilated or organ-perfusion support has been made and the discussion held with the next-of-kin, we contend that the potential donor’s end-of-life wishes regarding organ donation should be given due consideration. In the appropriate circumstances, the impact of DCD versus potential DBD pathways on the magnitude and nature of the resulting gift might be a reasonable component of the end-of-life discussion. Some families decide to discontinue mechanical, ventilated or organ-perfusion support as soon as possible but others encouragingly wait to fully honor the wishes of the dying potential donor, to maximize the opportunity of organ transplantation after brain death.

The data on marginal organs are compounded by the large and ever-growing concerns about post-transplantation outcomes [23,24]. Allograft and patient survival of DCD kidneys are reported to be similar to DBD kidneys, but DCD kidneys have been associated with increased resource utilization [24]. Saidi, *et al* [24], showed that ECD and DCD kidneys are associated with a higher frequency of hemodialysis need after transplantation, longer length of stay, more hospital readmissions due to poor or late-onset graft function and more cytomegalovirus infections in recipients of ECD and DCD kidneys, which resulted in a US$ 20,000–25,000 higher cost for their initial medical care and economic pressure on the transplant centers [24]. For DCD livers, there is a high rate of biliary strictures that have been attributed to the period of warm ischemia that occurs between withdrawal of donor life support and organ preservation. This leads to a reduction in graft survival and an increase in the need for retransplantation [23]. On the other hand, marginal liver allografts has been shown to be associated with increased hospital costs [25]. For heart, lung, and pancreas recipients, there is little utilization of DCD organs, though some centers have reported acceptable outcomes using DCD pancreata [26,27]. The concern about overall outcomes and cost of utilization of marginal organs can impact the decision of physicians and transplant centers to use these organs [24,25]. The transplant community must also monitor the effects of changes in organ procurement practices, especially defining optimal identification and management of marginal donors and more investment in live donation. There should also be emphasis on measurements to improve the quality of marginal organs such as *ex vivo* preservation methods or extracorporeal support for donors after cardiac death to assess viability and provide resuscitation of DCD and ECD organs [28,29]. A recent randomized trial have shown that protective ventilatory strategies such as low tidal volume can double the number of patients whose lungs were used for transplantation compared to conventional ventilatory methods [30]. Organ allocation and distribution have roots in the heterogeneous and somewhat arbitrary geographic boundaries that determine the current donation service areas and UNOS regions. This has led some to call for broader allocation units to make distribution more equitable and not based so tightly on geography. This can potentially lead to better utilization of organs and also decrease the discard rate. It has been shown that there was a wide variation in different regions regarding changes in organ recovery, transplantation and discards [31–33].

**UTILIZATION OF MARGINAL AND EXPANDED CRITERIA ORGANS (ECD)**

Our nationwide analysis confirmed a notable change in the diagnosis leading to donation over the past 10 years. There was a shift from trauma donors to donors with cardiovascular/cerebrovascular disease in the USA. The portion of donors who died of trauma decreased from 48.8% in era-1 (2001–2005) to 34.9% in era-2 (2006–2010). On the other hand, donors who died of cardiovascular disease/cerebrovascular disease rose from 38.1% in era-1 to 56.1% in era-2. The proportion of donors older than 64 years increased significantly from 6.9% in era-1 to 11.3% in era-2 and the proportion of donors with body mass index >35 kg/m^2^ also markedly increased from 6.8% to 11.2%. These also led to increased number of donors with medical comorbidities [14]. 

In addition to recent stagnant growth in overall donors, the percentage of standard criteria donor (SCD) steadily declined, from 78% in 1998 to about 65% in 2007 [1]. This decline can be attributed to increase in the number and percentage of ECDs and DCDs [1]. The shift in the distribution of recovered kidneys from SCD to ECD and DCD, impacts utilization, since DCD and ECD kidneys have higher rates of discard. The observed increase in DCD also explains, in part, the fewer organs per donor that are recovered and transplanted overall and the current state of less than 3.75 organs transplanted per donor, since it was 2.08 for DCD, 1.72 for ECD, and 3.63 for SCD in 2007 [34–36]. In 2007, 299 fewer SCD kidneys were transplanted (compared to 2006); there was an increase of 163 DCD non-ECD transplants [1–3,9,37]. Consistent with the goals set by Health Resources and Services Administration for DCD development, the percentage of donors from DCD continues to increase. There has been a total increase in the percentage of donors that are categorized as DCD, from 8% in 2006 to 9.8% in 2007, and the number and percentages of DCD liver and kidney transplants continue to increase substantially [1–3,9,37–42].

ECDs were defined by the UNOS criteria as all deceased donors age >60 years as well as those aged 50–59 years with at least two of the following comorbidities: i) history of hypertension, ii) cerebrovascular cause of brain death, or iii) terminal serum creatinine level >1.5 mg/dL. Donor kidney biopsy is regularly used in the evaluation of preexisting and terminal parenchymal pathology in ECD donors. A biopsy showing >20% glomerulosclerosis or moderate to severe tubular, interstitial or vascular changes is a contraindication to kidney utilization. ECD and DCD kidneys are usually placed on a pulsatile perfusion apparatus to potentially minimize preservation injury. Although pump parameters are not exclusively used to discard kidneys, a flow rate >80 mL/min and a resistance <0.40 mm Hg after a minimum of 6 hrs on the perfusion apparatus are considered reasonable thresholds for utilization.

Before the organ donation collaborative, the term ECD was used to classify subsets of deceased donor that are aged 60 years or older and those aged 50–59 years with at least two of the following characteristics: history of hypertension, serum creatinine level >1.5 mg/dL (132.6 µmol/L) and cerebrovascular cause of death [39]. These criteria define a donor population from whom the risk of graft failure after transplantation was anticipated to be 70% higher than after a non-ECD transplant [40–42]. Despite this expected higher rate of graft failure compared to SCD kidneys, multiple studies have subsequently shown that kidney transplantation using ECD is still associated with a substantial reduction in morbidity and improvement in life expectancy when compared with suitable transplant candidates who remained on maintenance dialysis treatment [40–42]. If we consider an increased risk of complications and death for those who have to wait, kidney transplants clearly save more lives and cost less as a treatment than does dialysis.

Ojo and colleagues showed that on average, recipients of ECD kidney transplants lived five years longer than transplant candidates who remained on dialysis, whereas SCD transplant recipients had a 13-year survival benefit [38]. Accordingly, ECD kidney transplants have continued to expand and comprise 15% of national deceased donor activity [1,2].

Another approach to the organ shortage has been the utilization of donors after cardiac death. The Institute of Medicine has studied the issues surrounding the use of DCDs, reaching the conclusion that “the recovery of organs from nonheart beating donors is an important, medically effective and ethically acceptable approach to reducing the gap that exists now and will continue to exist in future between the demand for and available supply of organs for transplantation” [40]. A number of investigators have reported excellent short-term outcomes using these donors, and several different organ procurement organizations have demonstrated 10%–15% growth in organ donation as a result of the use of DCD donors. Multiple studies have shown that the overall results of DCD (without ECD characteristics) and SCD kidney transplants are comparable [34–36,41–43].

Doshi, *et al*, reviewed the UNOS database (from 1998 to 2004) and showed that the 5-year allograft and patient survival rates of 66.9% and 81.3% in recipients of DCD kidneys was comparable with 66.5% and 80.8% graft and patient survival in the SCD group, respectively [43]. This most recent review also confirmed a higher incidence of delayed graft function in the DCD group compared to SCDs (41% *vs* 24%) which led to a longer length of hospital stay (10.2 *vs* 9 days). In addition, the incidence of rejection was similar in both groups (9.4% *vs* 10%). A study at the Massachusetts General Hospital, similarly, showed no differences in the long-term graft and patient survival, a higher incidence of delayed graft function, longer length of hospital stay and similar incidence of rejection in recipients of DCD versus SCD kidneys. Our long-term follow-up, however, reveals a more rapid attrition in survival of the ECD allografts (65% survival at 50 months *vs* 79%–80% for SCDs) [24].

Although we observed an increased incidence of delayed graft function using DCD and ECD kidney allografts, we found no difference in the long-term graft and patient survival between DCD and SCD recipients. This contrasts with other studies which have shown a negative impact of delayed graft function on kidney allograft survival from SCD donors [34–36,43–45]. Delayed graft function is a multifactorial phenomena resulting from warm and cold ischemia, poor donor quality and/or early rejection [45]. Since there was no correlation between delayed graft function and survival in our series, it might be postulated that our protocol of routine induction therapy with antithymocyte globulin and delayed tacrolimus introduction limited the likelihood of early subclinical rejection. This could provide an explanation for the lack of a detrimental impact of delayed graft function on long-term graft survival observed in our series. Of course, these observations require further studies to confirm the hypothesis.

Nationwide, a significant number of DCD and ECD kidneys continue to be discarded. However, as already noted, despite reports demonstrating that these kidneys have a higher rate of delayed graft function and a greater susceptibility to preservation injury as well as drug toxicity, the long-term outcome is quite satisfactory [46–53]. Thus, the major obstacle to more widespread utilization of these organs seems to be economic. We anticipated that the transplantation of ECD and DCD kidneys would result in higher costs when we embarked on utilizing these organs for our patients.

A recent study have documented the more frequent need for hemodialysis, longer length of hospital stay, more hospital readmissions due to poor or late onset graft function and more cytomegalovirus infections in recipients of ECD and DCD kidneys [24]. This resulted in a US$ 20,000–25,000 higher cost for their initial medical care [24]. Whiting, *et al*, have compared the economic costs of ECD kidney transplantation to those on hemodialysis [46]. They found the break-even point at which transplantation became less costly than ongoing hemodialysis ranged from 4.4 years for SCDs to as long as 13 years if an ECD kidney was transplanted into a high-risk recipient. The 5-year present value of payments for ECDs was significantly higher compared to SCDs: US$ 143,329 versus US$ 121,698, respectively. The average 1-year cost of hemodialysis for this cohort was US$ 28,666. They concluded that transplantation was always less expensive than hemodialysis. Although, in a strict cost analysis, ECD kidney recipients take longer to reach the break-even point and this point might not be reached consistently if allograft survival proved to be dramatically reduced in some patient cohorts, in general, utilization of DCD and ECD kidneys is still a cost-saving treatment strategy when compared to hemodialysis [54–56]. The cost for utilizing marginal donors is higher due to several factors discussed. Nevertheless, reimbursement for the transplantation services is based upon a single diagnostic-related group which assumes that all renal transplants require comparable resource utilization. In reality, the increased resource needs demonstrated by our study emphasize that centers who pursue the use of ECD and DCD kidneys are penalized financially for attempting to serve the largest possible number of patients with end-stage renal disease. As the transplant community seeks to utilize as many organs as possible in order to benefit increasing number of patients on the waiting list, we must take into consideration the true economic impact of such undertakings. This may not be addressed based on current remuneration schemes for services rendered. When costs increase significantly, as we have observed, transplant centers must absorb the deficit spending for each of these organs utilized. As a result, the current policies for reimbursement impose a significant burden on those transplant centers that accept these organs and jeopardize their financial viability despite the medical, social and ethical benefits of maximizing organ utilization. We advise, therefore, that a separate diagnostic-related group or modifier should be considered for ECD and DCD kidney recipients so that reimbursement is adjusted based on the donor and recipients risk. Such action can sustain the organ breakthrough collaboration efforts for increasing organ utilization. Otherwise, we risk ongoing underutilization of ECD and DCD allografts despite the documented benefits provided to end-stage renal disease recipients by these organs.

In conclusion, there is a general trend towards less ideal donors in the USA to expand the donor pool. This includes: 1) a decrease in the number of living donors; 2) an increase in deceased donors, primarily by an expansion in utilization of less ideal donors with chronic cardiovascular and cerebrovascular disease versus acute trauma victims; 3) increased use of more elderly and obese donors and a dramatic increase in DCD and marginal donors. These changes in practice have been associated with a reduction in the number of organs recovered and transplanted per donor, organs likely to have poorer function (ECD, DCD) and an increase in the discard rates. These changes are coincident with greater organ demand. The transplant community and policy makers should consider every option to expand the donor pool, avoid organ discards, and encourage the practices to optimize utilization of marginal organs. 
